# Dose-Response Met-RANTES Treatment of Experimental Periodontitis: A Narrow Edge between the Disease Severity Attenuation and Infection Control

**DOI:** 10.1371/journal.pone.0022526

**Published:** 2011-07-20

**Authors:** Carlos Eduardo Repeke, Samuel Barros Ferreira, Andreia Espindola Vieira, Elcia Maria Silveira, Mario Julio Avila-Campos, João Santana da Silva, Carlos Ferreira Santos, Ana Paula Campanelli, Ana Paula Favaro Trombone, Gustavo Pompermaier Garlet

**Affiliations:** 1 Department of Biological Sciences, School of Dentistry of Bauru, São Paulo University - FOB/USP, Bauru, Sao Paulo, Brazil; 2 Department of Microbiology, Institute of Biomedical Sciences, São Paulo University - ICB/USP, Sao Paulo, Sao Paulo, Brazil; 3 Department of Biochemistry and Immunology, School of Medicine of Ribeirão Preto, São Paulo University - FMRP/USP, Riberao Preto, Sao Paulo, Brazil; 4 Department of Pathology, Instituto Lauro de Souza Lima, Bauru, Sao Paulo, Brazil; University of Leuven, Rega Institute, Belgium

## Abstract

Chemokines and chemokine receptors have been implicated in the selective migration of leukocyte subsets to periodontal tissues, which consequently influences the disease outcome. Among these chemoattractants, the chemokines CCL3, CCL4 and CCL5 and its receptors, CCR1 and CCR5, have been associated with increased disease severity in mice and humans. Therefore, in this study we investigated the modulation of experimental periodontitis outcome by the treatment with a specific antagonist of CCR1 and 5 receptors, called met-RANTES. C57Bl/6 mice was orally infected with *Aggregatibacter actinomycetemcomitans* and treated with 0.05, 0.1, 0.5, 1.5 and 5 mg doses of met-RANTES on alternate days, and evaluated by morphometric, cellular, enzymatic and molecular methods. At 0.5 mg up to 5 mg doses, a strong reduction in the alveolar bone loss and inflammatory cell migration were observed. Interestingly, 5 mg dose treatment resulted in the maximum inhibition of inflammatory cell migration, but resulted in a similar inhibition of bone loss when compared with the lower doses, and also resulted in increased bacterial load and CRP response. When 0.5 and 5 mg therapy regimens were compared it was observed that both therapeutic protocols were able to downregulate the levels of pro-inflammatory, Th1-type and osteoclastogenic cytokines, and CD3+ and F4/80+ cells migration to periodontal tissues, but the high dose modulates host response in a more pronounced and unspecific and excessive way, interfering also with the production of antimicrobial mediators such as MPO, iNOS and IgG, and with GR1+ and CD19+ cells migration. Our results demonstrate a thin line between beneficial immunoregulation and impaired host defense during experimental periodontitis, and the determination of the exact equilibrium point is mandatory for the improvement of immune-targeted therapy of periodontitis.

## Introduction

The chronic immune response raised against periodontopathogens in periodontal environment result in the unremitting release of inflammatory mediators, which trigger soft and mineralized tissues destruction that typify periodontitis (PD) [1], [2]. Among inflammatory mediators present in PD development, chemokines are thought to play active roles in the development of host inflammatory immune reaction, which consequently can directly impact PD outcome from both tissue destruction and infection control viewpoints [3], [4].

Chemokines are small (8 to 14 Kd) proteins of cytokine family that coordinate the selective recruitment and function of specific leukocytes subsets at site of inflammation [5], [6]. Regarding PD, several chemokines have been found in inflamed periodontal tissues and implicated in disease pathogenesis [4], [7], [8], [9]. In fact, recent studies suggest that complex chemokine-chemokine receptors networks operate in periodontal tissues, such as the involvement of the chemokines CCL3, CCL4 and CCL5 in CCR1+ and CCR5+ cells migration and PD progression [10]. Therefore, the homologous chemokines CCL3, CCL4 and CCL5 share the specificity for binding the chemokines receptors CCR1 and CCR5, being primarily associated with chemoattraction of monocytic lineage (monocytes/macrophages and dendritic cells) through the binding to CCR1, while CCR5 also mediates the migration of lymphocytes polarized into Th1 phenotype [5], [6], [11]. Indeed, chemokine receptor CCR5 mediates chemoattraction of pro-osteoclastic and osteoclastogenic leukocyte subsets throughout experimental PD [12], reinforcing the role of chemokine system in PD pathogenesis. Therefore, while Th1-polarized lymphocytes and macrophages cells are characteristic sources of bone resorptive and inflammatory cytokines, cells of the monocytic lineage are potentially prone to develop an osteoclastic phenotype, which may directly impact the bone resorption activity [10], [13], [14], [15].

Given the important role of chemokines in a wide range of inflammatory diseases, including bone diseases such as rheumatoid arthritis, they became interesting targets for therapeutic intervention [11], [16]. While classic anti-inflammatory drugs impair the mobilization of leukocyte subsets in a non-selective way, the development of chemokine/chemokine receptors inhibitors allows a targeted intervention with potential higher benefits and lower side effects [11], [17]. In fact, while some leukocyte subsets are potentially destructive during chronic inflammation, certain subsets are fundamental in the control of infection processes and in the tissue healing and repair [17], [18]. Therefore, the search for anti-inflammatory and immunomodulatory for intervention in periodontal diseases ideally must consider both host defense and tissue destruction viewpoints.

Previous studies demonstrate that the absence of the chemokine receptors CCR1 and CCR5 result in an attenuated PD phenotype in mice [10], [19], suggesting a potential for therapeutic intervention. Also, preliminary data demonstrate that the simultaneous blockade of such receptors with a specific antagonist, called met-RANTES, result in a higher effectiveness in the inhibition of inflammatory cell influx and alveolar bone loss [10]. Met-RANTES is the resultant of the recombinant CCL5 (previously called RANTES) modification by the extension of the product with a single methionine residue, which does not compromise the binding to the cognate receptors but impair the subsequent signaling and cellular response [20]. In this way, Met-RANTES is effective in the attenuation of several inflammatory diseases including rheumatoid arthritis, which share with PD characteristics as the chronic nature of inflammatory response and the bone resorptive activity [11], [20], [21], [22], [23], [24], [25].

However, while preliminary data suggests a potential application of Met-RANTES as a therapeutic strategy in order to control PD [10], the ideal therapeutic protocol from the dose-response viewpoint, the mechanisms involved in the virtual attenuation of PD severity, as well the potential side-effects in the control of periodontal infection remain unknown. In this study, we investigated effectiveness of met-RANTES treatment in the control of experimental PD in mice by means of a dose-response approach, where tissue destruction and infection markers were monitored and the possible mechanisms by which met-RANTES modulates disease outcome were investigated by cellular, enzymatic and molecular methods.

## Materials and Methods

### Experimental groups

Experimental groups comprised 8-week-old male C57BL/6 wildtype (WT) mice, bred and maintained during the experimental period in the animal facilities of School of Dentistry of Bauru, São Paulo University - FOB/USP. Throughout the period of the study the mice were fed with sterile standard solid mice chow (Nuvital, Curitiba, PR, Brazil) and sterile water. Experimental groups comprised 12 mice (5 for both flow cytometry and alveolar bone loss analysis, 3 for RealTimePCR and 4 for ELISA) as described previously [10], [26]. Data from one experiment representative of two is depicted in the graphs and described in the results section. The experimental protocol was approved by the Institutional Committee for Animal Care and Use of the School of Dentistry of Bauru, São Paulo University, protocols #015/2007 & #016/2007.

### Periodontal infection and Met-RANTES treatment

Bacterial culture and periodontal infection were performed as described previously [10], [26]. In brief, the animals received an oral delivery of 1×10^9^ colony-forming units (CFU) of a diluted culture of *A. actinomycetemcomitans* JP2 (grown anaerobically in supplemented agar medium, TSBV), in 100 µl of phosphate-buffered saline (PBS) with 2% of carboxymethylcellulose, placed in the oral cavity of mice with a micropipette. After 48 and 96 h, this procedure was repeated. The treatment with met-RANTES (Serono Pharmaceutical Institute of Research, Switzerland) was performed as previously described [10], [27], [28], consisting in an intraperitoneal injection of 0.05, 0.1, 0.5, 1.5 and 5 mg/kg of met-RANTES diluted in PBS, on alternate days, simultaneously initiated with the induction of PD protocol until day 30 post-infection, when the samples were collected. The control groups consisted of non-infected mice, treated or not with met-RANTES and infected mice treated or not with vehicle (PBS).

### Quantification of alveolar bone loss

Evaluation of the extent of alveolar bone loss was performed as described previously [10], [26]. The maxillae were hemisected, exposed overnight in 3% hydrogen peroxide and defleshed mechanically. The palatal faces of the molars were photographed at 20× magnification using a dissecting microscope (Leica, Wetzlar, Germany), with the occlusal face of the molars positioned perpendicularly to the base. The images were digitized and analyzed using ImageTool 2·0 software (University of Texas Health Science Center, San Antonio, TX, USA). Quantitative analysis was used for the measurement of the area between the cement–enamel junction (CEJ) and the alveolar bone crest (ABC) in the three posterior teeth, in mm^2^. At day 30 post-infection, five animals were analyzed, and for each animal the alveolar bone loss was defined as the average of CEJ–ABC between the right and the left arch; data are presented from one experiment that is representative of two.

### Isolation of inflammatory cells from periodontal tissues and flow cytometric analysis

The isolation and characterization of leukocytes present in the lesion site were performed as described previously [10], [26]. The whole buccal and palatal periodontal tissues of upper molars were collected, weighed and incubated for 1 h at 37°C, dermal side down, in RPMI-1640, supplemented with NaHCO_3_, penicillin/streptomycin/gentamycin and 0.28 Wunsch units/ml of liberase blendzyme CI (Roche–F. Hoffmann-La Roche Ltd, Basel, Switzerland). The tissues of five mice were processed in the presence of 0·05% DNase (Sigma- Aldrich, Steinhein, Germany) using Medimachine (BD Biosciences PharMingen, San Diego, CA, USA), according to the manufacturer's instructions. After processing, cell viability was assessed by Trypan blue exclusion, and the cell count was performed in a Neubauer chamber. Flow Cytometry analysis of leukocyte surface markers and intracellular cytokines was then performed in fixed and permeabilized cells with Cytofix/Cytoperm (BD Biosciences), according to the manufacturer's instructions. The cells were labeled (4°C/20 min) with the optimal dilution of PE/FITC-conjugated antibodies against F4/80, CD3, Gr1 and CD19 staining antibodies, as well as with respective isotype controls (BD Biosciences PharMingen), and analyzed with a FACS (FACScan & CellQuest software, BD Biosciences). The absolute and relative numbers of leukocyte subsets were calculated based on the total amount of cells determined in the Neubauer chamber count and the number of stained cells obtained by FACS. Results represent the number of cells ± SD in the periodontal tissues of each mouse, normalized by the tissue weight, for two independent experiments.

### Protein extraction and ELISA

Measurements of cytokine in homogenized palatal periodontal tissue were performed by ELISA as previously described (12), using commercially available kits (R & D Systems, Taufkirchen, Germany), as follows: IL-1β (sensitivity >3 pg/ml), TNF-α (>3–4 pg/ml), IL-6 (1.8 pg/ml), IL-10 (>4 pg/ml), IFN-γ (>2 pg/ml), IL-4 (>2pg/ml), IL-17 (1.6 pg/ml) and RANKL (<3.9 pg/ml). Data from one experiment representative of two is presented in the results.

### Serum C reactive protein (CRP) measurement

The levels of serum CRP were determined using a commercially available agglutination kit (Labtest Diagnóstica, São Paulo, Brazil). In brief, 50 ml of serum samples (diluted 4, 16, 64, 128 and 256 times), 50 ml of 0·9% NaCl and 50 ml of a solution containing latex beads coated with anti-CRP antibodies were dispensed in 96-well plates. The plate was agitated with circular movements for 2 min, and the macroscopic evidence of agglutination was observed. For the semiquantification of CRP levels, the level of assay sensitivity (>6 mg/l) were multiplied by the titre of CRP of each sample. One experiment representative of two is presented in the results.

### Periodontal tissue MPO activity

The activity of MPO in periodontal tissue was measured as described previously [29]. Briefly, periodontal tissues were homogenized in ice-cold buffer (0·1 MNaCl, 20 mMNaPO4, 15 mN Na EDTA), pH 4·7, and centrifuged at 3000 *g* for 15 min. The pellet was then subjected to hypotonic lysis (900 ml of 0·2% NaCl solution for 30 s followed by addition of an equal volume of a solution containing 1·6% NaCl and 5% glucose). After further centrifugation, the pellet was resuspended in 50 mM NaPO4 buffer, pH 5·4, containing 0·5% hexadecyltrimethylammonium bromide (H-TAB) and rehomogenized. The homogenate was then frozen and thawed three times and centrifuged again at 10 000 *g* for 15 min at 4°C. MPO activity in the resuspended pellet was assayed by measuring the change in absorbance at 450 nm using tetramethylbenzidine (1·6 mM) and H2O2 (0·5 mM). A unit of MPO activity was defined as that converting 1 mmol of hydrogen peroxide to water in 1 min at 22°C. Based in control samples and previous experiments the assay limit detection are MPO 25 as measured at 490 nm DO. One experiment representative of two is presented in the results.

### Serum antibody to *A. actinomycetemcomitans*

The levels of serum antibody specific to *A. actinomycetemcomitans* were measured as described previously [29]. Briefly, 96-well microtitre plates (Corning Incorporated, Corning, NY, USA) were coated with formalin-fixed whole bacterial cells in 0·1 M sodium carbonate buffer, incubated at room temperature for 4 h, washed three times with PBS (0·05% Tween 20) and then blocked with PBS containing 5% bovine serum albumin (BSA) for 30 min. Serial serum dilutions were added, incubated at room temperature for 2 h and, after washing the wells three times, 100 ml of peroxidaseconjugated anti-mouse IgG (Zymed; Invitrogen Life Technologies, Carlsbad, CA, USA) were added as a detection antibody. After washing three times, 100 ml of substrate buffer (o-phenylenediamine dihydrochloride) was added and incubated for 45 min. The enzymatic reaction was stopped by adding 50 ml of 3 N HCl and the absorbances were measured at 492 nm with a microtitre plate reader (EMAX; Molecular Devices Corporation, Sunnyvale, CA, USA). Non-infected mice serum was used as a control for non-specific binding. The IgG levels are expressed as the antibody serum titre; based in control samples and previous experiments the assay limit detection for Aa-specific IgG is 0.35 as measured at 490 nm DO. One experiment representative of two is presented in the results.

### Real-time PCR reactions

Real-time PCR was used to analyze the levels of Aa DNA and iNOS mRNA in periodontal tissues. The extraction of total RNA (performed with Trizol reagent, Invitrogen, Rockville, MD, USA) or DNA (performed with DNA Purification System, Promega Biosciences Inc., San Luis Obispo, CA, USA) from periodontal tissues were accomplished as described previously [29]. Both procedures were performed from a sample comprising the upper molars with their alveolar bone, and the whole surrounding buccal and palatal periodontal tissues, which was frozen in liquid nitrogen, mechanically fragmented and homogenized in sterile Milli-Q water with Ultra Turrax (IKA, Germany), and subsequently submitted to DNA or RNA extraction. Real-time PCR quantitative mRNA or DNA analyses were performed in MiniOpticon system (BioRad, Hercules, CA, USA) using the SybrGreen system (Applied Biosystems, Warrington, UK). SybrGreen PCR MasterMix (Applied Biosystems), 100 nM specific primers (designed with the software Primer Express 3.0 from Aplied Biosystems, Foster City, CA, USA; and subsequently synthesized by Invitrogen; iNOS - sense: CGTCATTTCTGTCCGTCTCT; anti-sense: TTGCTGGCTGATGGCTGGCG; and *A. actinomycetemcomitans* JP2 - Sense: ATGCCAACTTGACGTTAAAT; anti-sense: AAACCCATCTCTGAGTTCTTCTTC; β-actin - sense: ATGTTTGAGACCTTCAACA; anti-sense: CACGTCAGACTTCATGATGG) and 2.5 ng of cDNA or 5 ng of DNA in each reaction [10]. The standard PCR conditions are were 95°C (10 min), followed by 40 cycles of 94°C (1 min), 56–65°C (1 min) and 72°C (2 min), and by the standard denaturation curve. For RNA analysis, gene expression levels were determined using the Ct method and normalized by housekeeping gene beta-action, while for Aa DNA quantification, in the view of the absence of an internal control (or a standard curve for absolute quantification), DNA levels were determined using the Ct method with reference to a positive control of Aa DNA from culture and then normalized by the tissue weight to avoid technical variances and interferences in the results. Negative controls without cDNA/DNA and without reverse transcriptase were also performed; data are presented from one experiment that is representative of two.

### Statistical analysis

Data are presented as means ± SD, and the statistical significance between the infected and control groups of WT and met-RANTES treated mice was analyzed by ANOVA, followed by Bonferroni post test, or by the unpaired t-test, both performed with GraphPad Prism 5.0 software (GraphPad Software Inc., San Diego, CA). Values of P<0.05 were considered statistically significant.

## Results

### Dose response analysis of met-RANTES treatment effect in experimental PD outcome

**Figure 1:**
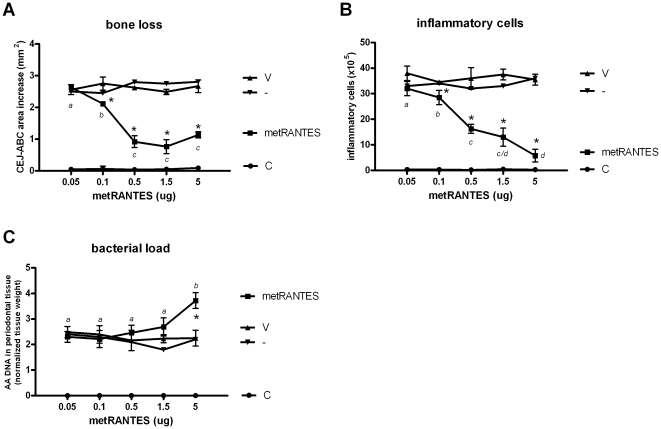
The effect of met-RANTES treatment at different doses in the modulation of alveolar bone loss, inflammatory cell influx and bacterial load in experimental periodontal disease. C57Bl/6 mice were infected orally with *A. actinomycetemcomitans* and treated with met-RANTES at 0.05, 0.1, 0.5, 1.5 and 5 mg doses or (V) veicule, (-) non treated and (C) control non-infected mice and evaluated at 30 day post-infection for: A) alveolar bone loss quantification, performed through the measurements of cement-enamel junction-alveolar bone crest (CEJ-ABC) area in the palatal face of maxillary molars; B) total leukocyte counts of inflammatory infiltrate, performed in a Neubauer chamber; and C) *A. actinomycetemcomintans* load (AA DNA) in periodontal tissues, quantified by Real-TimePCR, using SybrGreen System and the cycle threshold (Ct) method; all performed as described in the Material and methods; *p<0.05 versus non-treated mice, unpaired t-test. Different italic low case letters represent statistically significant differences among the doses in the same experimental group (P<0.05; One-way ANOVA).

Our first aim was to determine the dose of met-RANTES which result in maximum decrease of experimental PD severity parameters (Fig. 1). When compared to untreated and vehicle-treated mice, met-RANTES doses of 0.1, 0.5, 1.5 and 5 mg were found to significantly inhibit both alveolar bone loss and inflammatory cell influx. When the distinct doses were compared, the 0.1 mg doses was found to be less effective than 0.5, 1.5 and 5 mg in the attenuation of bone loss while no significant differences were found between these three higher doses. Regarding inflammatory cell counts, our results demonstrate that as a general rule the dose increase resulted in a gradual decrease of inflammatory cell influx into periodontal tissues, except for non-significant differences between the doses of 0.5 and 1.5, and 1.5 and 5 mg. When the bacterial load was evaluated, our results demonstrated that only the 5 mg dose interfered in the control of infection, as revealed by the significant increase in AA DNA levels in periodontal tissues when compared to untreated, vehicle-treated and to the other met-RANTES doses groups. During the experimental period the mice mortality was absent and no significant change in mice weight among the experimental groups was verified (data not shown). Our results demonstrate that 10X dose range resulted in distinct modulation of experimental PD outcome from the tissue destruction and infectious viewpoints, and therefore the doses of 0.5 and 5 mg were selected for the subsequent evaluations.

### Phenotypic analysis of leukocytes migration interference by distinct doses of met-RANTES treatment

**Figure 2:**
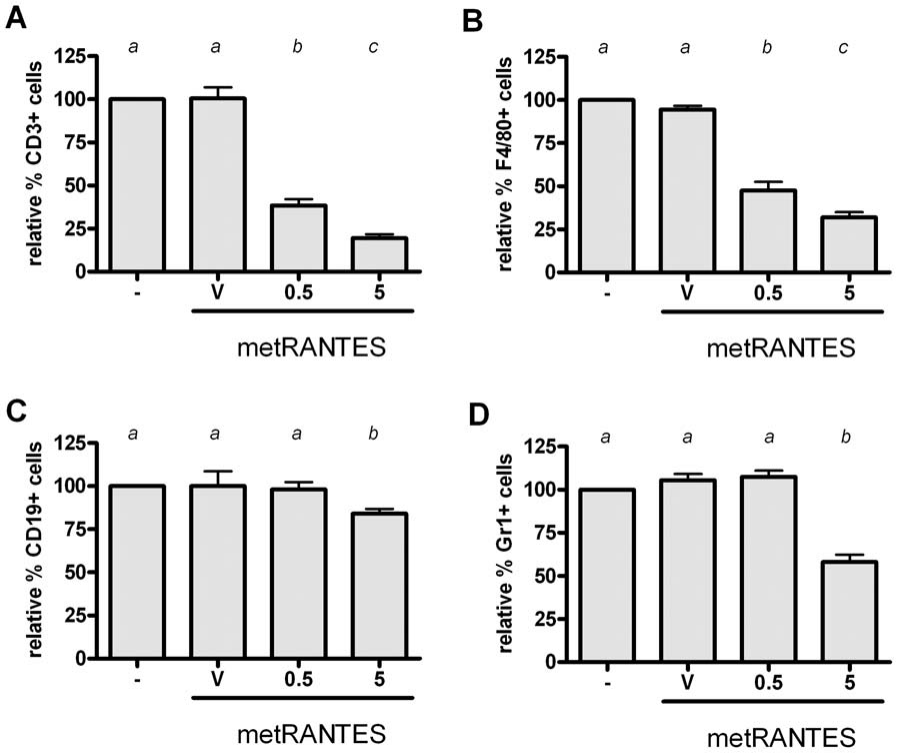
Phenotypic analysis of leukocytes migration interference by distinct doses of met-RANTES treatment. C57Bl/6 mice were infected orally with *AA*, treated with met-RANTES at 0.5 and 5 mg doses or (V) veicule and (-) non treated, sacrificed at day 30 post-infection and evaluated for relative number of: A) F4/80+; B) CD3+; C) Gr1+; and D) CD19+ cells in periodontal tissues of WT non-treated versus vehicle, 0.5 mg and 5 mg met-RANTES treated mice by flow cytometry, all performed as described in Material and methods. Results demonstrate the relative number of cells in % when compared with untreated group. Different italic low case letters represent statistically significant differences among the doses in the same experimental group (P<0.05; One-way ANOVA), statistical analysis performed with raw data before % conversion.

In order to determine the mechanisms underlying the differential response to 0.5 and 5 mg met-RANTES doses, we initially investigated the interference of the distinct doses in the migration of F4/80+, CD3+, Gr1+ and CD19+ cells (Fig. 2). The 0.5 mg dose resulted in a significant reduction in the relative number of F4/80+ and CD3+ cells, while no differences was observed in the reduction of relative number of Gr1+ and CD19+ cells when compared with non-treated and vehicle-treated mice. Differently, the 5 mg met-RANTES protocol reduced the relative % of F4/80+, CD3+, Gr1+ and CD19+ cells in the periodontal tissues when compared with 0.5 mg of MetRANTES.

### The distinct dose-response modulation of cytokine milieu in periodontal tissues by met-RANTES

**Figure 3:**
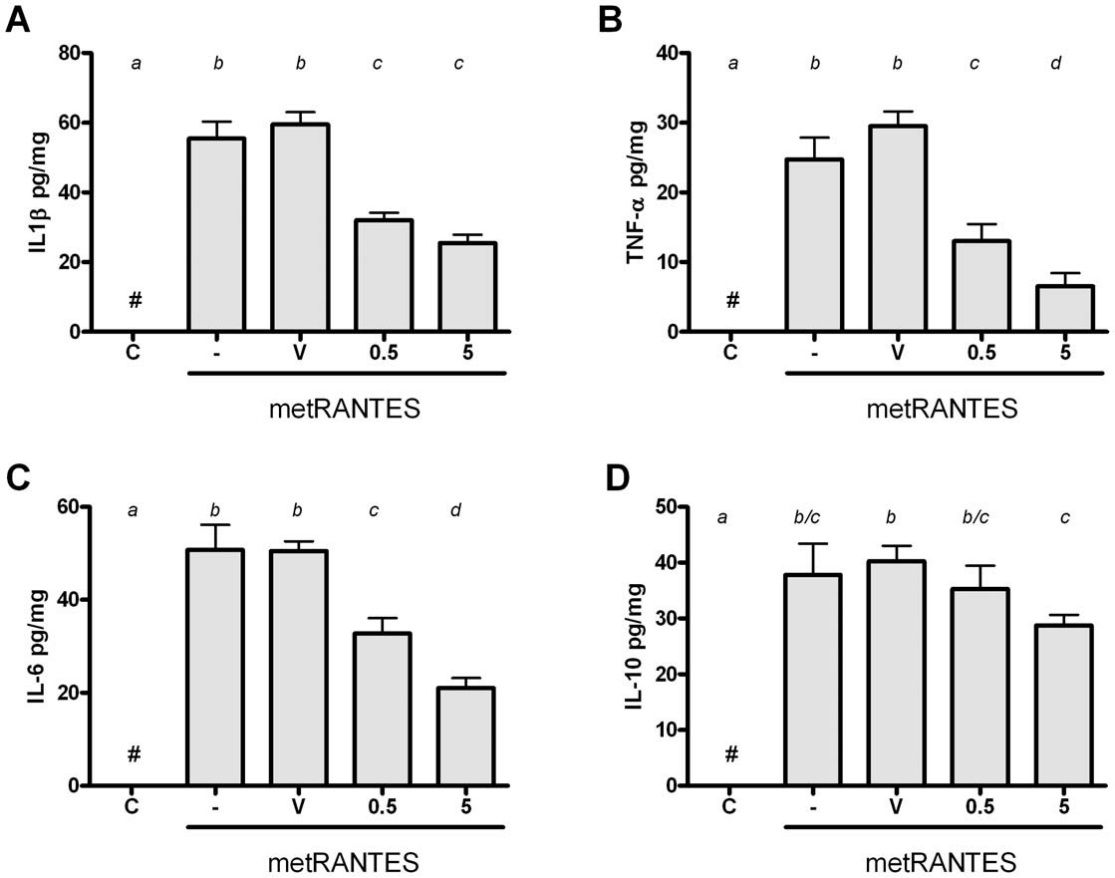
Met-RANTES treatment at different doses modulate pro- and anti- inflammatory cytokine levels in experimental periodontal disease. C57Bl/6 mice were infected orally with *AA*, treated with met-RANTES at 0.5 and 5 mg doses or (V) veicule, (-) non treated and (C) control non-infected mice, sacrificed at day 30 post-infection and evaluated for levels of A) IL-1β; B) TNF-α; C) IL-6 and D) IL-10 cytokines. Different italic low case letters represent statistically significant differences among the doses in the same experimental group (P<0.05; One-way ANOVA).

**Figure 4:**
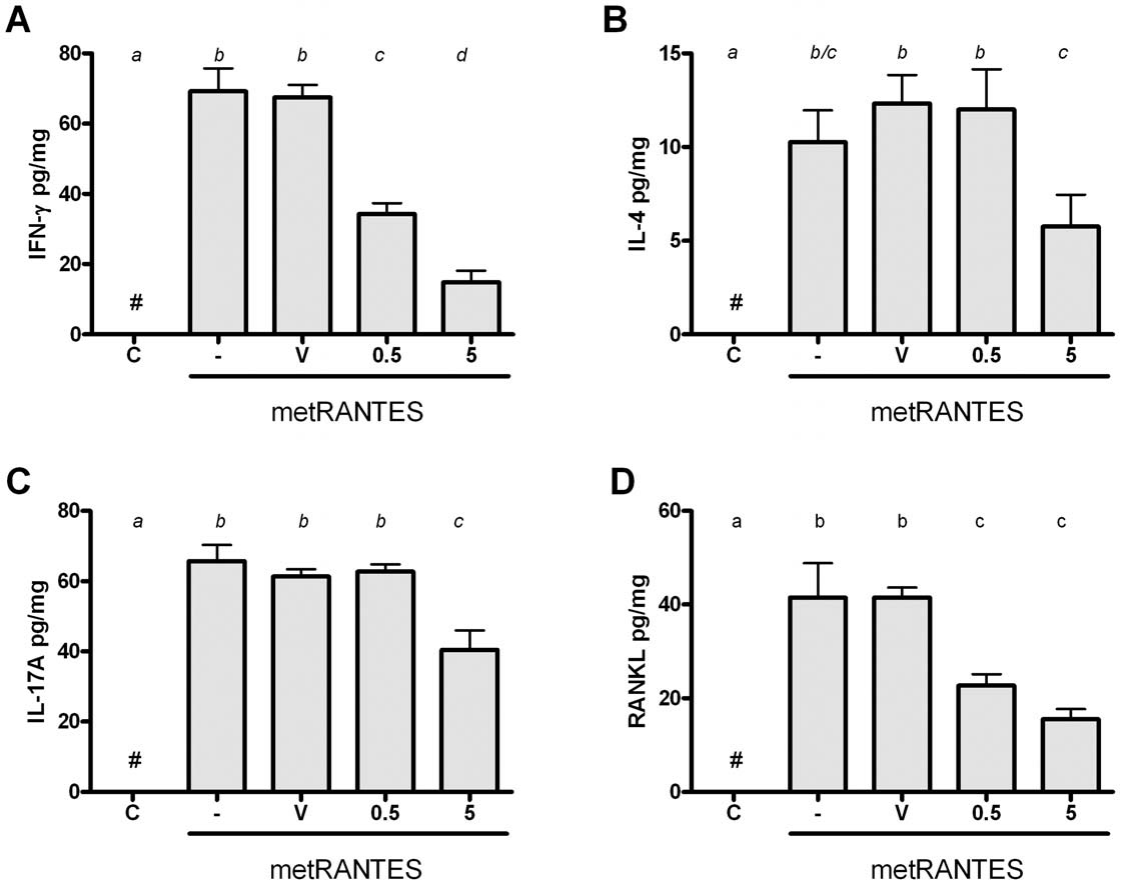
Met-RANTES treatment at different doses modulate Th1, Th2, Th17 cytokine and osteclastogenic factor RANKL levels in experimental periodontitis. C57Bl/6 mice were infected orally with *AA*, treated with met-RANTES at 0.5 and 5 mg doses or (V) veicule, (-) non treated and (C) control non-infected mice, sacrificed at day 30 post-infection and evaluated for levels of A) IFN-γ, a Th1 cytokine; B) IL-4, a Th2 cytokine; C) IL-17A, a Th17 cytokine and D) the major osteoclastogenic factor RANKL. Different italic low case letters represent statistically significant differences among the doses in the same experimental group (P<0.05; One-way ANOVA).

In order to determine the mechanisms underlying the differential response to 0.5 and 5 mg met-RANTES doses, we next analyzed the levels of several cytokines in periodontal tissues of mice by ELISA (Fig. 3). Our results demonstrate that, as a general rule, both 0.5 and 5 mg were able to decrease the production of proinflammatory cytokines IL-1β, TNF-α and IL-6 in periodontal tissues, but in different degrees, since 5 mg dose resulted in a higher decrease of TNF-α and IL-6 when compared to the 0.5 mg dose. Complementarily, our data demonstrate that only the 5 mg dose resulted in a significant modulation of the anti-inflammatory cytokine IL-10 when compared to the other experimental groups. When T helper-signature cytokines were evaluated (Fig. 4), it was observed that IFN-γ levels were significantly reduced by both 0.5 and 5 mg when compared to controls, but this change was significantly more prominent with the 5 mg dose. Regarding IL-4 and IL-17A levels, only the 5 mg dose resulted in a decrease in the level of such cytokines in the periodontal tissues of AA-infected mice. Finally, we observed that both 0.5 and 5 mg treatment resulted in decreased levels of RANKL in periodontal tissues at the same extent, when compared to the controls.

### The distinct dose-response modulation of antimicrobial response by met-RANTES therapy

**Figure 5:**
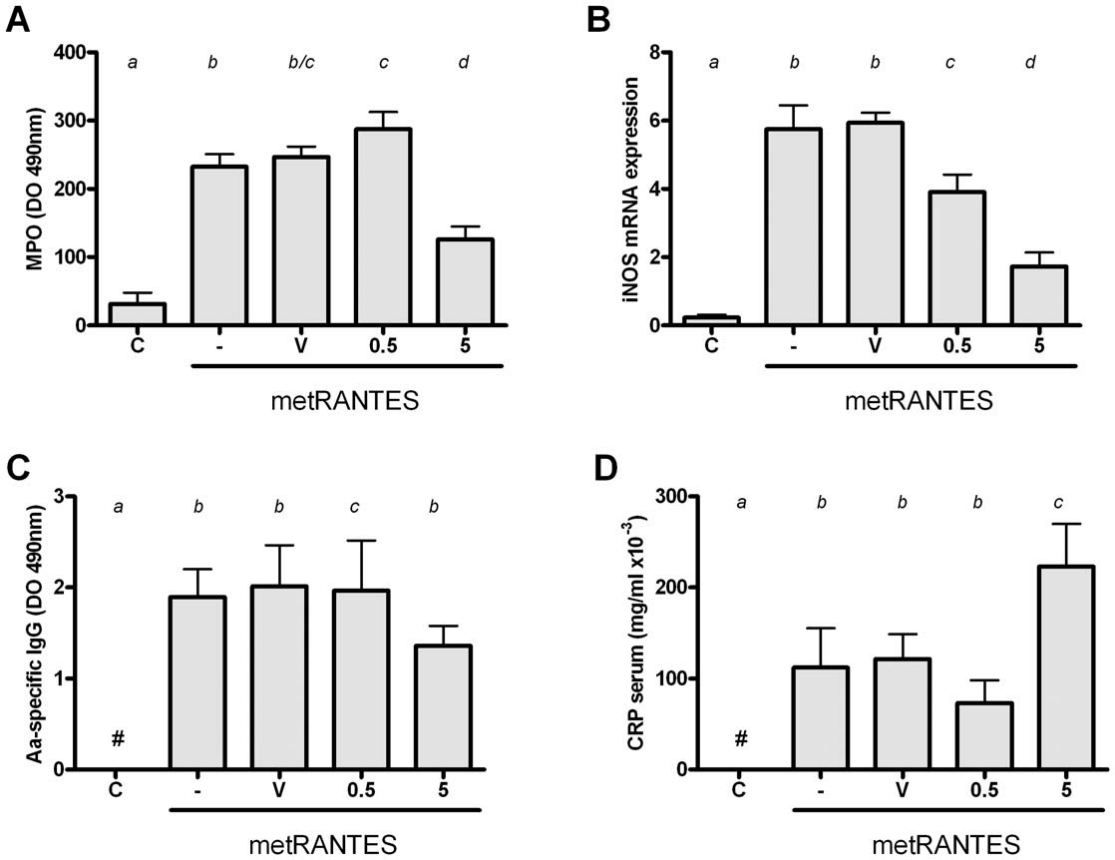
The effect of distinct met-RANTES doses treatment in the control of *A. actinomycetemcomitans* infection. C57Bl/6 mice were infected orally with *AA*, treated with met-RANTES at 0.5 and 5 mg doses or (V) veicule, (-) non treated and (C) control non-infected mice, sacrificed at day 30 post-infection and evaluated for: A) levels of myeloperoxidase (MPO) in periodontal tissues; B) levels of inducible nitric oxide syntase (iNOS) expression; C) levels of Aa-specific IgG; and D) levels of C reactive protein (CRP) in serum; all performed as described in Material and methods. Different italic low case letters represent statistically significant differences among the doses in the same experimental group (P<0.05; One-way ANOVA).

In the view of the distinct effect of 0.5 and 5 mg met-RANTES doses in the bacterial load in periodontal tissues, we next investigated the levels of antimicrobial factors and the systemic infection parameter C reactive protein (CRP) (Fig. 5). Our results demonstrate that the levels of the antimicrobial factor and PMN marker MPO in periodontal tissues were differentially modulated by the distinct met-RANTES doses, while 0.5 mg dose resulted in a slight (but significant) increase of MPO levels, the 5 mg dose resulted in a lower level of MPO activity in periodontal tissues than controls. Regarding the levels of iNOS mRNA, our results demonstrate that iNOS expression in periodontal tissues was significantly reduced by both 0.5 and 5 mg when compared to controls, but a major downregulation was verified with the 5 mg dose. When the levels of serum AA-specific IgG were investigated, our results demonstrate that only 5 mg met-RANTES dose resulted in a decrease of antibody levels. Finally, our data demonstrate that the levels of systemic inflammation marker CRP were slightly decreased (non-statistically significant) by the 0.5 mg met-RANTES dose, while the 5 mg dose resulted in a significant increase in serum CRP when compared with the other experimental groups.

## Discussion

PD is characterized by the chronic host response triggered by periodontopathogens that result in soft and mineralized tissues destruction [1], [2]. Among the host mediators involved in the generation and maintenance of this exacerbated inflammatory immune response, chemokines and chemokine receptors have been implicated in PD development [3], [4], [30]. The chemokines receptors CCR5 and CCR1 are supposed to mediate the chemoattraction of lymphocytes polarized into Th1 phenotype and monocytes/macrophages into periodontal tissues, which consequently contributes to disease progression [3], [4], [8], [19]. Preliminary results demonstrate that a specific blocker of CCR5 and CCR1 chemokine receptors, called met-RANTES, was able to decrease the experimental PD severity [10]. However the mechanisms involved in such modulation, the ideal dose of met-RANTES to achieve the maximum remission of experimental PD, as well potential side effects concerning the infectious aspects of the disease remain unknown.

Therefore, we initially performed a dose-response (5 doses with a 100X range, 0.05 to 5 mg) analysis of experimental PD outcome following met-RANTES therapy. The alveolar bone loss and inflammatory cell influx were used as the disease severity parameters, and the bacterial load in the periodontal tissues was used to monitor the control of infection. Except for the 0.05 mg dose, all the other doses (0.1 up to 5 mg) resulted in a significant decrease of experimental PD severity. Interestingly, as a general rule a dose-response effect was identified in the attenuation of inflammatory cells influx, while a similar inhibition of bone resorption was achieved with the doses ranging from 0.5 up to 5 mg. The effectiveness of the met-RANTES in the control of inflammation-associated tissue damage is in agreement with previous studies [22], but deeper comparisons with other models are impaired by the relatively low number of previous studies, performed in quite different experimental models usually investigating one or at most two different met-RANTES doses within a narrow interval [31], [32], [33]. Interestingly, the PD treatment with 5 mg of met-RANTES dose impaired the control of periodontal infection, evidencing to distinct situations, where similar protective effects from the tissue destruction viewpoint are associated with distinct (normal and impaired) infectious outcomes. Therefore, in order to investigate the mechanisms underlying such differential response, the experimental groups receiving met-RANTES doses of 0.5 and 5 mg were selected for the further analysis.

The first scenario, represented by the 0.5 mg met-RANTES protocol, can be interpreted as a clinically desired situation, where the tissue damage was minimized without interfering with host defense process. In fact, the 0.5 mg met-RANTES dose was effective in reducing F4/80 (a monocytic lineage marker) and CD3 cells (CD3 lymphocytes), which present CCR1 and CCR5 receptor in its membranes, and also was observed a reduction in the tissue levels of pro-inflammatory cytokines IL-1β, TNF-α and IL-6. These cytokines are classically implicated in the onset and progression of PD by its role in osteoclastogenic process [2], [34], [35], being the reduction in its levels compatible with the attenuation of the experimental disease severity observed. Accordingly, met-RANTES therapy target cells from the monocytic lineage, characteristic sources of IL-1β, TNF-α and IL-6 [33]. Also in agreement with our findings, met-RANTES treatment also reduced TNF-α and IL-1β levels in a rat arthritis model [31], [33]. In addition, 0.5 mg dose of met-RANTES was able to reduce CD3+ cells in the periodontal tissues, which potentially include Th1-polarized lymphocytes, the main source of IFN-γ, which can also justify the downregulation of the IFN-γ levels observed. In spite of some controversies, IFN-γ is present at high levels in periodontal lesions and associated with higher PD severity [29], [36]. Th1 cells are also supposed to be a significant source of the major osteoclastogenic factor RANKL in periodontal environment [12], [37]. Accordingly, met-RANTES therapy also resulted in a significant downregulation of RANKL levels, which also could account for a direct effect in the inhibition of bone resorptive process [38].

Interestingly, the levels of IL-10, IL-4 and IL-17A were not modulated by the 0.5 mg met-RANTES treatment, suggesting a specific effect over determined leukocyte subsets. Also, met-RANTES treatment at 0.5 mg dose does not interfere in the migration of Gr1 and CD19 positive cells to periodontal tissues, reinforcing the specificity of the leukocyte subsets targeted by met-RANTES. However, it is also possible that met-RANTES specifically modulate adaptive immunity through the interference with dendritic cell traffic from peripheral tissues to lymphoid organs, which involves the receptor CCR5 [39]. It is also important to consider that even in the total absence of CCR1 and CCR5 function (which is probably not fully achieved *in vivo* even with substantially high met-RANTES doses), a remaining migration of monocytic lineage cell and Th1 lymphocytes is still expected and possibly mediated by other chemokine receptors, such as CCR2 and CXCR3, respectively [40]. This redundancy in the chemokine system possibly account for the residual (but significant) IFN-γ and pro-inflammatory cytokine production verified in the animal treated with met-RANTES. Interestingly, when considered from the infectious viewpoint, the 0.5 mg met-RANTES treatment protocol was found to not interfere in the control of experimental infection (as evidenced by the similar bacterial load in periodontal tissues and CRP serum levels), reinforcing therefore a potential clinical application of met-RANTES to treat PD.

However, in the second scenario identified, the increase of met-RANTES dose to 5 mg in spite of an additional reduction in the inflammatory cell influx, does not result in further inhibition of bone loss and impair host defense. Concerning the more pronounced effect of the 5 mg dose in the inhibition of inflammatory cell influx, this group presented lower levels of TNF-α, IL-6, IFN-γ and IL-17 when compared to the group with received 0.5 mg of met-RANTES, which could account for the additional reduction in cell migration observed, not only in the total amount of inflammatory cells but also in the population of macrophages and CD3 lymphocytes. However, regarding the comparable bone loss levels presented by both groups, it is possible that the similar levels of RANKL in the tissues are responsible for the equivalent bone resorption degree. Interestingly, the levels of IL-17A and IL-4, theoretically products of Th17- and Th2-polarized lymphocytes, were also reduced by the 5 mg met-RANTES dose. Since Th17 and Th2 helper subsets specifically migrate using the chemokine receptors CCR6 and CCR4/CCR8 [5], [41], not targeted by met-RANTES, our data demonstrate that the higher dose result in a broader and probably unspecific modulation of host response, not observed with the 0.5 mg dose. One possible explanation for such phenomenon is that the high met-RANTES doses could interfere indirectly in the cell migration mediated by other receptor than CCR1 and CCR5. In fact, our data demonstrate a reduction in the number of CD19 and Gr1 positive cells, presumably B cells and neutrophils respectively, cells which normally do not present CCR1 and CCR5 receptors, by 5 mg dose of met-RANTES. Chemokine receptor blockade by met-RANTES limits the endothelial cell-leukocyte interaction, which consequently can limit the migration process even if mediated by non-blocked receptors [42]. Also, the continuous administration of a high amount of met-RANTES may result in physical competition with other chemokines for binding sites in the extracellular matrix (ECM) [43]. In fact, the stable binding of chemokines to the glycosaminoglycan side chain of ECM proteoglycans generates a chemotactic gradient required for the directional cell migration [43]. Therefore, the predominance of met-RANTES distribution over the ECM would impair or limit the presence of other chemoattractants and consequently result a broader interference in chemotaxis process. However, the glycosaminoglycan-binding properties of met-RANTES remain unknown. An additional hypothesis is that the more pronounced modulation of cytokine production by the high met-RANTES doses impact as a cascade the sequential immune response, modulating differentially the local production of other chemokines that consequently impact the subsequent response.

The broad and unspecific immunomodulation promoted by the high met-RANTES doses is also perceptible by the analysis of the host antimicrobial response. Indeed, our data demonstrate that MPO and IgG levels were only affected by the 5 mg dose treatment. MPO and IgG are products of neutrophils and B cells respectively, cells types supposedly to not be primarily affect by the interference with CCR1 and CCR5 promoted by met-RANTES [10]. In accordance with such hypothesis, 5 mg dose of met-RANTES treatment reduced the number of both Gr1+ and CD19+ cells in the infected periodontal tissues. In addition, it is important to mention that the expression of the antimicrobial enzyme iNOS was also greatly diminished by the 5 mg met-RANTES dose. Taken together, the reduction in the innate and adaptive antimicrobial response is possible responsible for the increase in the bacterial load found in periodontal tissues. Accordingly, MPO, iNOS and IgG are supposed to contribute to the killing of periodontal bacteria [26], [29], [44], [45]. Therefore, it is possible to conclude that an excessive downregulation of host response by the high met-RANTES doses significantly impair host response overcoming the protective effect presented by lower doses. In fact, the significant increase in serum CRP levels reinforces the compromised host response presented by mice treated with high met-RANTES doses.

In this setting, an important question refers to nature and intensity of host response required to assure a proper defense against the periodontopathogens. Since a series of therapeutic proposals targets the modulation of host response, the exact determination of the leukocyte subsets, cytokines and antimicrobial factors really involved in the control of periodontal infection is fundamental to direct future host-targeted therapies. When the results presented here are compared with a previous study of our group, were a mice strain selected for minimal inflammatory response (AIRmin) was evaluated [23], interesting information arise. AIRmin strain and the C57BL/6 mice treated with 5 mg met-RANTES dose present similar inflammatory and bone loss scores, and also similar production of IL-1β and TNF-α [23]. However, AIRmin strain efficiently controls the experimental periodontal infection, a finding associated with higher IFN-γ production than met-RANTES-treated mice [23]. Also, while MPO and iNOS levels were similar in AIRmin strain and met-RANTES-treated mice, 5 mg met-RANTES treatment resulted in lower IgG response than observed in AIRmin strain [23]. In accordance with previous studies [29], [45], these results point to important roles of IFN-γ and antibody production in the control of periodontal infection [2]. Therefore, it is possible to suggest that immunoregulatory strategies aimed to control PD should not interfere with these mediators. However, it is important to consider that a highly complex host-pathogen interaction takes place in the periodontal environment, and that the exact nature and intensity of the “ideal” protective host response remain to be determined. Also in this context, it is important to mention that while PD and rheumatoid arthritis are often compared in the view of the similar chronic nature of inflammatory response and the bone resorptive activity; the theoretical sterile condition of arthritis versus the infective nature of PD result in more complex scenario in the case of PD, where an additional factor is added to an already intricate system.

In summary, our results demonstrate that the therapeutic efficacy of met-RANTES treatment of experimental PD is dose-dependent. While low to intermediate doses are effective in attenuating inflammatory cell migration and bone loss via downregulation of the local level of pro-inflammatory, Th1-type and osteoclastogenic cytokines, high doses seem to modulate host response in a unspecific and excessive way, resulting a more pronounced modulation of cytokine production and interfering in the host antimicrobial response. This results points to a potential role of met-RANTES in the clinical management of PD, but reinforce the requirement of detailed and careful investigation of the degree of host response aimed in the view of potential side-effects associated.
